# Exploring temporal transcription regulation structure of *Aspergillus fumigatus *in heat shock by state space model

**DOI:** 10.1186/1471-2164-10-306

**Published:** 2009-07-08

**Authors:** Jin Hwan Do, Rui Yamaguchi, Satoru Miyano

**Affiliations:** 1 Human Genome Center, Institute of Medical Science, University of Tokyo, Shirokanedai, Minato-ku, Tokyo 108-8639, Japan

## Abstract

**Background:**

The thermotolerance of *Aspergillus fumigatus *plays a critical role in mammalian and avian infections. Thus, the identification of its adaptation mechanism to higher temperature is very important for an efficient anti-fungal drug development as well as fundamental understanding of its pathogenesis. We explored the temporal transcription regulation structure of this pathogenic fungus under heat shock conditions using the time series microarray data reported by Nierman *et al*. (*Nature *2005, 438:1151-1156).

**Results:**

The estimated transcription regulation structure of *A. fumigatus *shows that the heat shock proteins are strongly negatively associated with central metabolic pathway genes such as the tricarboxylic acid cycle (TCA cycle) and carbohydrate metabolism. It was 60 min and 120 min, respectively, after the growth temperature changes from 30°C (corresponding to environments of tropical soil) to 37°C and 48°C (corresponding to temperatures in the human body and compost, respectively) that some of genes in TCA cycle were started to be upregulated. In these points, most of heat shock proteins showed lowest expression level after heat shocks. Among the heat shock proteins, the HSP30 (AFU6G06470), a single integral plasma membrane heat shock protein, presented most active role in transcription regulation structure in both heat shock conditions of 37°C and 48°C. The metabolic genes associated with multiple genes in the gene regulation network showed a tendency to have opposite expression patterns of heat shock proteins. The role of those metabolic genes was second regulator in the coherent feed-forward loop type of regulation structure having heat shock protein as its first regulator. This type of regulation structure might be very advantageous for the thermal adaptation of *A. fumigatus *under heat shock because a small amount of heat shock proteins can rapidly magnify their regulation effect on target genes. However, the coherent feed-forward loop type of regulation of heat shock proteins with metabolic genes became less frequent with increasing temperature. This might be the reason for dramatic increase in the expression of heat shock proteins and the number of heat shock response genes at heat shock of 48°C.

**Conclusion:**

We systemically analysed the thermal adaption mechanism of *A. fumigatus *by state space model with times series microarray data in terms of transcription regulation structure. We suggest for the first time that heat shock proteins might efficiently regulate metabolic genes using the coherent feed-forward loop type of regulation structure. This type of regulation structure would also be efficient for adjustment to the other stresses requiring rapid change of metabolic mode as well as thermal adaptation.

## Background

*Aspergillus fumigatus *is both a primary and opportunistic pathogen as well as a major allergen associated with severe asthma and sinusitis [1]. The host respiratory system, including that of human, is constantly exposed to its spore because of prolific production of spores and nearly ubiquitous distribution in the environment, at a density of 1 to 100 conidia/m^3 ^[2]. The spores can be easily eliminated by the innate immune system from lung epithelial tissue in immunocompetent vertebrates. However, immunocompromised individuals are at risk of invasive aspergillosis as a consequence of proliferation on pulmonary or other tissues via mycelial growth. Invasive aspergillosis has an associated mortality rate ranging from 50 % to 90 % depending on the patient population [3]. The prosperity of *A. fumigatus *in mammalian and avian infection depends basically on its thermotolerance [4]. Therefore, the identification of the adaptation mechanism of this fungus to higher temperature is very important for efficient anti-fungal drug development as well as fundamental understanding of pathogenesis of *A. fumigatus*.

Nierman *et al*. [4] examined the gene expression change throughout a time course upon shift of growth temperatures from 30°C to 37°C and 48°C for investigation of the metabolic adaptation of *A. fumigatus*. They suggested that host temperature alone (37°C) is insufficient to turn on many virulence-related genes because no known genes implicated in pathogenicity showed higher expression at 37°C than that at 48°C. Lamarre *et al*. reported another genome-wide expression study of this fungus at 37°C with different age of conidia [5]. Their microarray data suggested that dormancy is associated with fermentation and reduced metabolism. In both studies, genome-wide expression profiles were well analysed from a simple level including identification of differentially expressed genes to some complex level including classification of genes with similar profiles, but the transcription regulation structure between genes in a system level has been never studied. Actually, the regulation of gene expression is achieved through genetic regulatory systems structured by networks of interactions between DNA, RNA, proteins, and small molecules [6]. Thus, it is important to understand how genes in *A. fumigatus *are transcriptionally regulated for its thermal adaptation.

Several methods have been proposed for modelling gene networks including Boolean networks [7,8], Bayesian networks [9], differential equations [10] and state space model (SSM) [11,12]. Especially, the temporal network structure of gene regulatory mechanism can be facilitated with time-course microarray data. However, a wide variety of statistical models proposed for estimation of temporal gene network structure are frequently limited because the length of the time-course data is fairly short, e.g. typically less than 10 whereas the number genes involved ranges from 10^2 ^to 10^4^. To overcome such a limitation, Hirose *et al*. [12] proposed a module-based gene network estimation which explores genetic networks of the transcriptional modules which are sets of genes sharing a common function or involved in the same path way rather than the use of gene level networks. The transcriptional modules may be considered as the groups of transcriptionally co-expressed genes. These transcriptional modules can be again mapped onto the gene-level networks. Here, we employed this module-based approach for the estimation of temporal transcription regulation structure of *A. fumigatus *in heat shock with microarray data consisting of very short length of time point, *i.e*., 6. The time series microarray data reported by Nierman *et al*. [4] were used in this study. We also compared the temporal transcription regulation structures in two different heat shock conditions, *i.e*., shift from 30°C to 37°C and 48°C, respectively.

## Results

### Metabolic response of *A. fumigatus *to heat shock

**Figure 1:**
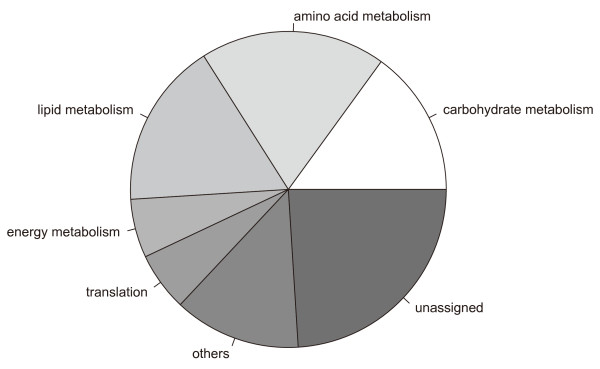
**The metabolic distribution of common genes (596 genes) between heat shock response genes of 37°C and 48°C**.

To reduce the effect of missing values in time series microarray data of *A. fumigatus *in heat shock condition, the genes with over 11 % of missing values in total observations were excluded (see Methods section for detail). Thus, 4027 and 4771 genes from 9,516 genes on the array were first chosen in this study for heat shock of 37°C and to 48°C, respectively. From these two gene lists, we selected the significantly differentially expressed genes showing over two fold change in expression level and below 0.05 of *p*-value in *t*-test for at least a time point. Here, we will call these genes heat shock response genes. Finally, the number of heat shock response gene became 726 and 2200 for heat shock of 37°C and 48°C, respectively. Our study was focused on these heat shock response genes. Eighty two percent (596 genes) of heat response genes at 37°C were also observed in the set of heat response genes at 48°C. Almost half these common genes were involved in the central metabolic pathways such as carbohydrate, amino acid and lipid metabolism (Figure 1).

**Figure 2:**
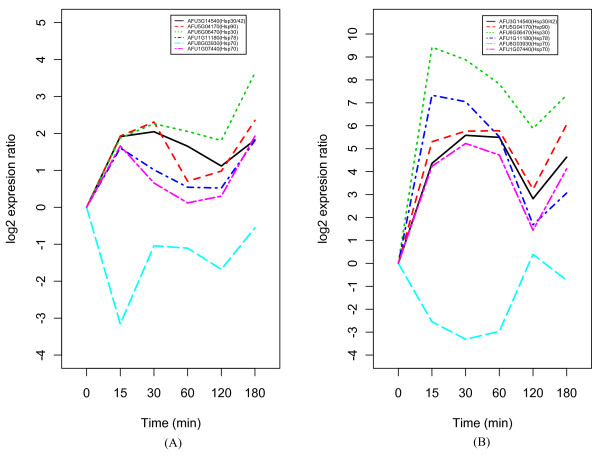
**The average gene expression profiles of heat shock proteins**. (A) expression profiles of heat shock proteins at 37°C, (B) expression profiles of heat shock proteins at 48°C.

For the more detail analysis, we examined the response of each metabolic pathway in each time point for these common heat response genes (see the additional file 1). The metabolic pathways showing the high sensitivity at the first time point (15 min) after heat shock were nucleotide metabolism including purine and pyrimidine and ribosome involved protein synthesis. Especially, the response genes involved in ribosome were almost down-regulated throughout the time course in both heat shocks of 37°C and 48°C. The citrate cycle (TCA cycle) genes were initially downregulated, but many of these genes were upregulated from 60 min and 120 min, respectively, after the heat shock of 37°C and 48°C. There are many metabolisms with positively regulated genes at 60 min after the heat shock of 37°C including glycolysis/gluconeogenesis, fructose/mannose metabolism, fatty acid metabolism, and amino acid metabolism including glycin, serine, threonine, histidine, phenylalanine, tyrosine and tryptophan. Similar trend was also observed at 120 min after the heat shock of 48°C for metabolisms including pyruvate and amino acids such as valine, leucine and isoleucine as well as TCA cycle. This might be related to the decrease of expression level of heat shock proteins other than AFU8G03930 (Hsp70) at 60 min and 120 min, respectively, after heat shock of 37°C and 48°C (Figure 2). Considering overall metabolic response to heat shock, the heat shock of 48°C seems to bring more severe metabolic disturbance to the cells of *A. fumigatus *than that of 37°C.

### Identification of transcriptional module in heat shock response of *A. fumigatus *using state space model

**Figure 3:**
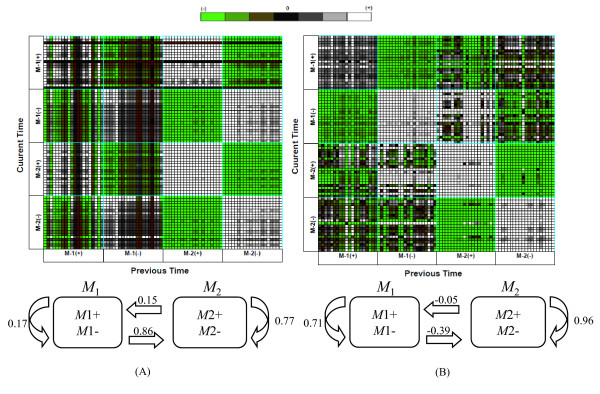
**The heat map of estimated temporal relationship between top-ranked 20 genes in each sub-module and temporal relationship between modules**. The upper part represents the heat map of temporal relationship from previous time to current time between top ranked 20 genes in each sub-module. The gene pairs associated with strong negative regulation are presented as deep green while the white regions represent gene pairs with strong positive regulation. The lower part shows the temporal relationship between modules and the number on arrow represents regulation coefficient estimated by system model (equation 3 in Methods section). (A) heat shock of 37°C, (B) heat shock of 48°C.

We first identified the potential transcriptional modules, sets of genes that are co-regulated under heat shock condition, to map them on the gene-level network by state space model (SSM) (see Methods section for detail). Each module is assumed to follow the states of a first-order Markov chain and a gene may belong to more than one module. The dimension of the state variable *x*_*n *_was chosen by *k *= 2 in this study (see Methods section). The regulation relationship between sub-modules are more clear than that between modules (the upper part of Figure 3). We selected top-ranked 15 genes from all sub-modules including *M*_1+_, *M*_1-_, *M*_2+ _and *M*_2- _(Table 1). The plus (+) and minus (-) module in each module usually have opposite expression pattern for each other. In heat shock of 37°C, four heat shock proteins including AFU6G06470 (Hsp30), AFU5G07340 (Hsp40), AFU3G14540 (Hsp30/Hsp42) and AFU1G15270 (Hsp98/Hsp104) appeared in *M*_1+ _while the other heat shock protein AFU8G03930 (Hsp70) was in *M*_1-_. This assignment of heat shock proteins to each sub-module shows a good agreement with their expression patterns (Figure 2). The expression pattern of AFU8G03930 (Hsp70) is unusual compared with those of other Hsp70s including AFU1G07440 (Hsp70) and AFU2G04620 (Hsp70) (see the additional file 2 for expression profiles of AFU1G07440 and AFU2G04620). This suggests that the AFU8G03930 might have different function from general Hsp70 which plays an important role in normalizing proteins degenerated by stress, and in maintaining the physiological functions of cells.

**Table 1:** The list of the top-ranked 15 genes in each sub-module.

**Module**	**Gene**	**Heat shock at 37°C**	**Gene**	**Heat shock at 48°C**
1(+)	AFU6G06470	Heatshock protein Hsp30-like, putative	AFU1G10130	Adenosylhomocysteinase
1(+)	AFU6G12160	C6 transcription factor, putative	AFU2G03610	IMP dehydrogenase, putative
1(+)	AFU7G05580	Phospholipase PldA, putative	AFU5G04220	Mitochondrial DNA replication protein
1(+)	AFU1G11220	hypothetical protein	AFU5G14660	GABA permease, putative
1(+)	AFU2G04020	Alpha, alpha-trehalose phosphate synthase subunit, putative	AFU4G07360	Cobalamin-independent methionine synthase MetH/D
1(+)	AFU6G00420	hypothetical protein	AFU7G01220	Farnesyl-diphosphate farnesyltransferase, putative
1(+)	AFU5G07340	DnaJ domain protein Psi, putative	AFU5G14650	RING finger protein
1(+)	AFU1G17020	UDP-Glucose dehydroGenase	AFU3G13400	nucleolar protein nop5
1(+)	AFU3G14540	Heatshock protein Hsp30/Hsp42, putative	AFU6G09990	Importinbeta-4subunit, putative
1(+)	AFU5G13750	Calcium binding protein Caleosin, putative	AFU3G03290	hypothetical protein
1(+)	AFU1G15270	Heat shock protein Hsp98/Hsp104/ClpA, putative	AFU5G05710	Centromere/microtubule-binding protein cbf5
1(+)	AFU4G13630	hypothetical protein	AFU6G11890	Dynamin GTPase, putative
1(+)	AFU3G01440	conserved hypothetical protein	AFU1G15900	Importinbeta-2subunit, putative
1(+)	AFU6G00410	amino acid permease, putative	AFU8G03930	Hsp70 chaperone
1(+)	AFU1G17010	Glutathione S-transferase, putative	AFU1G05990	RibosomalproteinL16a

1(-)	AFU7G05460	Putative uncharacterized protein	AFU6G06470	Heat shock protein Hsp30 like, putative
1(-)	AFU2G03610	IMP dehydrogenase, putative	AFU1G11180	Heat shockprotein/chaperonin HSP78, putative
1(-)	AFU6G09990	Importin beta-4subunit, putative	AFU3G00960	conserved hypothetical protein
1(-)	AFU1G06190	Histone H4 arginine methyltransferase RmtA	AFU3G12630	related to CYTOCHROME B561
1(-)	AFU1G15620	ATP-dependent RNA helicase HelA	AFU6G10470	Zinc finGer protein ZPR1
1(-)	AFU5G05710	Centromere/microtubule-bindinGprotein cbf5	AFU2G03060	Cactin
1(-)	AFU7G03690	Protein pxr1	AFU6G12160	C6 transcription factor, putative
1(-)	AFU8G03930	Hsp70 chaperone	AFU2G10910	MFS alpha-glucoside transporter, putative
1(-)	AFU3G13400	nucleolar protein nop5	AFU5G04170	Heat shock protein 90
1(-)	AFU6G10320	WD repeat protein; possible nuclear pore complex associated	AFU3G14550	RNA polymeraseII transcription factor B subunit 5
1(-)	AFU3G09600	sik1 protein	AFU4G06220	conserved hypothetical protein
1(-)	AFU3G10800	Eukaryotic translation initiation factor 3 subunit CLU1/TIF31, putative	AFU2G00210	hypothetical protein
1(-)	AFU1G09770	La domain family	AFU1G15270	Heat shock protein Hsp 98/Hsp 104/ClpA, putative
1(-)	AFU7G02610	hypothetical protein	AFU4G11620	DNA replication complex GINS protein psf3
1(-)	AFU1G09200	Ribosome bioGenesis protein Erb1, putative	AFU4G08810	unnamed protein product

2(+)	AFU6G08760	Proline oxidase PrnD	AFU7G01000	Aldehyde dehydrogenase, putative
2(+)	AFU6G08750	Delta-1-pyrroline-5-carboxylate dehydrogenase PrnC	AFU6G11430	Aldehyde dehydrogenase AldA, putative
2(+)	AFU6G07720	Phosphoenolpyruvate carboxykinase AcuF	AFU5G08930	Isovaleryl-CoA dehydrogenase IvdA, putative
2(+)	AFU3G11790	Galactose-proton symport, putative	AFU6G03590	Methyl citrate synthase precursor
2(+)	AFU3G14590	Copper amine oxidase, putative	AFU4G12010	2-oxoacid dehydrogenases acyltransferase, putative
2(+)	AFU6G03730	2-methyl citrate dehydratase, putative	AFU6G03060	MFS monosaccharide transporter, putative
2(+)	AFU6G02860	Isocitratelyase	AFU6G03730	2-methylcitrate dehydratase, putative
2(+)	AFU4G11310	Fructose-1,6-bisphosphatase Fbp1, putative	AFU6G02860	Isocitrate lyase
2(+)	AFU7G01000	Aldehyde dehydrogenase, putative	AFU3G01450	3-methyl-2-oxobutanoate dehydrogenase, putative
2(+)	AFU6G14200	Acetyl-CoA-acetyltransferase, putative	AFU6G06470	Heatshock protein Hsp30 like, putative
2(+)	AFU1G00410	cutinase transcription factor 1 beta	AFU3G09690	thaumatin-like protein, putative
2(+)	AFU2G00210	hypothetical protein	AFU3G00960	conserved hypothetical protein
2(+)	AFU4G12010	2-oxoacid dehydrogenases acyltransferase, putative	AFU5G04170	Heatshock protein 90
2(+)	AFU8G04130	C6 transcription factor	AFU6G08760	Prolineoxidase PrnD
2(+)	AFU4G08170	Succinate-semialde hydedehydrogenase Uga2, putative	AFU2G02050	Peptidyl-prolylcis-transisomerase D

2(-)	AFU6G10650	ATP citrate lyase, subunit1, putative	AFU4G06890	14-alphasterol demethylase Cyp51A
2(-)	AFU6G10660	ATP citrate lyase subunit	AFU1G14930	hypothetical protein
2(-)	AFU1G03150	C-14 sterol reductase	AFU7G05060	MGtC/SapB family membrane protein
2(-)	AFU1G17630	FAD/FMN-containing protein	AFU2G00320	Sterol delta5,6-desaturase, putative
2(-)	AFU5G02330	Ribonuclease mitogillin precursor	AFU3G14920	TAM domain methyltransferase, putative
2(-)	AFU4G11720	Phosphatidyl synthase	AFU1G03150	C-14 sterol reductase
2(-)	AFU3G08580	Glycine-rich RNA-binding protein, putative	AFU8G05990	hypothetical protein
2(-)	AFU2G00320	Sterol delta5,6-desaturase, putative	AFU4G14250	hypothetical protein
2(-)	AFU3G07230	Phosphopantothenate-cysteine ligase, putative	AFU5G02330	Ribonuclease mitogillin precursor
2(-)	AFU3G03580	Transferase family protein	AFU7G05490	hypothetical protein
2(-)	AFU3G10750	Acetate kinase, putative	AFU5G13730	invasion associated protein p60
2(-)	AFU6G07900	Rod1 protein	AFU5G06240	Alcohol dehydrogenase, putative
2(-)	AFU4G06890	14-alpha sterol demethylase Cyp51A	AFU1G16190	Probable glycosidase crf1 precursor
2(-)	AFU3G08990	unnamed protein product	AFU5G03800	High-affinity ironpermease
2(-)	AFU7G04730	major facilitator protein homolog, putative	AFU7G05500	Glutathione S-transferase, putative

The heat shock proteins with similar expression pattern are assigned to same module. The average expression profiles of all heat response genes are shown at additional file 2.

The regulation relationships among modules are shown in the lower part of Figure 3. The number of above arrow represents the strength of regulation while the positive and negative value indicates positive and negative regulation, respectively. Module *M*_1 _containing heat shock proteins shows strong positive association with module *M*_2 _including many pathway genes in heat shock of 37°C while the module *M*_2 _including many pathway genes in heat shock of 48°C represents the strong positive self-regulation. This indicates that the regulation of metabolic gene by heat shock proteins might be stronger at heat shock of 37°C than at heat shock of 48°C.

### Transcription regulation structure of heat shock response genes in *A. fumigatus*

**Figure 4:**
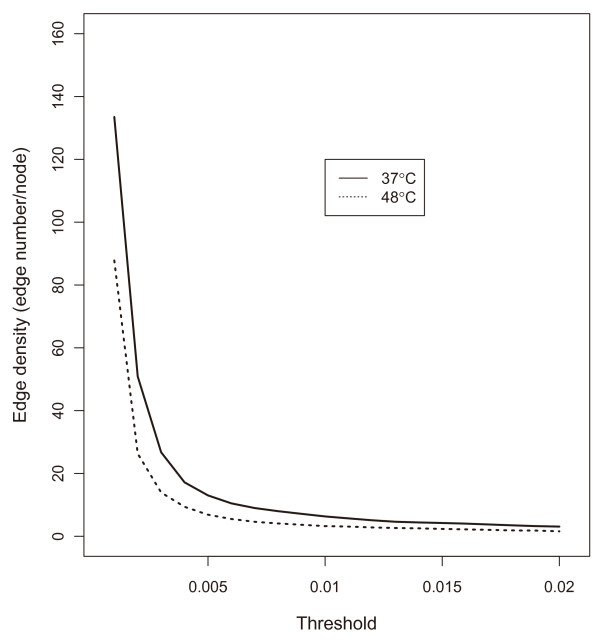
**The dependence of edge density on threshold in networks of 37°C and 48°C**.

**Figure 5:**
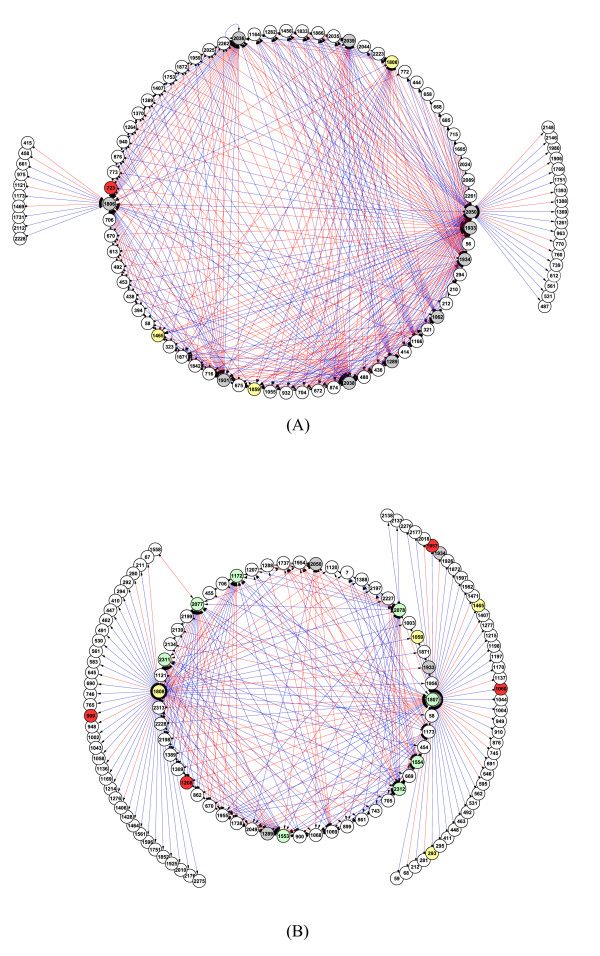
**The visualization of estimated transcriptional network at the threshold of 0.015**. The node in the network stands for gene. The yellow and red nodes represent heat shock proteins and transcription factors, respectively. The hub node is shown as grey and green node, respectively, at network of 37°C and 48°C. The red and blue arrows represent positive and negative regulation, respectively. The direction of arrow indicates the regulation direction, *i*.*e*., from regulator gene to regulated or target gene. The gene information corresponding to yellow node (heat shock protein): 293 (AFU1G11180), 1059 (AFU3G14540), 1465 (AFU5G04170), 1807 (AFU6G06460), 1808 (AFU6G06470). The gene information corresponding to red node (transcription factor): 723 (AFU3G02000), 909 (AFU3G09670), 1060 (AFU3G14550), 1208 (AFU4G09710), 1957 (AFU6G12160). The gene information corresponding grey and green nodes (hub nodes) : 1062 (AFU3G14590), 1172 (AFU4G08340), 1553 (AFU5G08750), 1554 (AFU5G08800), 1806 (AFU6G06430), 1289 (AFU4G12010), 1931 (AFU6G10610), 1933 (AFU6G10650), 1934 (AFU6G10660), 2038 (AFU7G00170), 2036 (AFU7G00120), 2039 (AFU7G00200), 2050 (AFU7G01000), 2077 (AFU7G01920), 2078 (AFU7G01930), 2311 (AFU8G06340), 2312 (AFU8G06350). (A) network of 37°C, (B) network of 48°C. Gene information corresponding to each node in the network is shown at additional file 3.

We estimated the transcription regulation structure by mapping the identified transcriptional modules in the above section onto the gene-level networks via the first order vector autoregression form (see Methods section for detail). The edge density in the network of heat shock 37°C is always higher than that of 48°C over the threshold range surveyed (Figure 4). The reduction of edge density in higher temperature was reported by Takemoto *et al*. [13]. They have suggested that with increasing temperature the metabolic networks undergo a change from heterogeneous and high-modular structures to homogeneous and low-modular structures like random networks. This suggests that the cells of *A. fumigatus *in the heat shock of 48°C might have less dense metabolic network than those in heat shock of 37°C. In our model, each element of the autoregressive coefficient matrix stands for interaction strength of corresponding gene pair (see Methods section for detail). Thus, the higher value it has, the stronger interaction the gene pair has. We can construct a sparse biological network by choosing a threshold. In this study, we have chosen a threshold of 0.015 from the region showing almost constant edge density in both heat shock conditions of 37°C and 48°C (Figure 4). Therefore, we obtained the two networks, one is composed of 418 edges and 99 genes at heat shock of 37°C and another consists of 308 edges and 132 genes at the heat shock of 48°C (Figure 5). The gene information corresponding to each node is shown at additional file 3. The red and blue arrows represent positive and negative regulation, respectively. The direction of arrow indicates the regulation direction, *i.e*., from regulator gene to regulated or target gene. Except for heat shock protein, the gene with more than twenty edges is called hub gene in this study which is indicated by grey and green node, respectively, in the networks of 37°C and 48°C. Three heat shock proteins including 1059 (AFU3G14540, Hsp30/42), 1465 (AFU5G04170, Hsp90) and 1808 (AFU6G06470, Hsp30) appeared in both networks of 37°C and 48°C while 293 (AFU1G11180, Hsp78) is observed only at network of 48°C. The gene number associated with the 1808, a single integral plasma membrane heat shock protein, dramatically increased at network of 48°C. This suggests that the 1808 might have more critical role in the high temperature than in low temperature.

**Figure 6:**
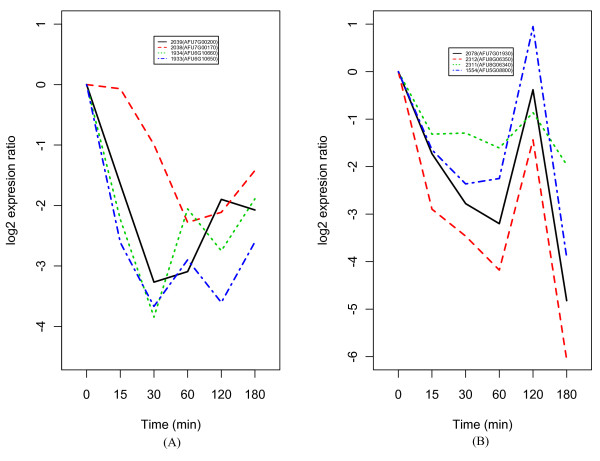
**The average expression profiles of hub nodes negatively regulated by heat shock proteins**. (A) expression profiles of hub nodes at network of 37°C, (B) expression profiles of hub nodes at network of 48°C.

From the network, we can estimate possible regulation between heat shock response genes. For example, the regulation of 561 (AFU2G04010, *α*-*α* trehalose phosphate synthase subunit) by 1808 in network of 48°C shows the possible regulation of trehalose production during heat shock condition. Trehalose is known to accumulate at high levels to stress conditions [14]. The positive regulation of trehalose metabolism by 1808 shows a good agreement with upregulation of AFU3G12100 (trehalose synthase) during time course at both heat shock conditions (see additional file 2). Many metabolic genes are targeted by heat shock proteins (Table 2). It is interesting to notice that some of metabolic genes are playing the role as key regulator such as heat shock protein 1808 (Figure 5). Most of these metabolic genes represent clear contrast in their expression patterns with heat shock proteins and play role as hub nodes in the network (Figure 6). In terms of regulation, these metabolic genes seem to help the regulation of heat shock proteins. That is, the metabolic genes such as 1933 (AFU6G10650), 1934 (AFU6G10660), 2038 (AFU7G00170) and 2039 (AFU7G00200) tend to positively regulate the genes negatively regulated by heat shock protein 1808 at network of 37°C (Figure 5A). The positive regulation of these metabolic genes towards target genes brings out the same effect as negative regulation by heat shock proteins towards the same target genes. This is mainly due to the opposite expression pattern between the metabolic gene and heat shock protein 1808. This coherent type of regulation could make it possible to maximize the regulation efficiency of heat shock proteins. The metabolic genes with similar function also appeared at the network of 48°C including 2312 (AFU8G06350) and 2078 (AFU7G01930) (Figure 5B).

**Table 2:** The genes showing association with heat shock proteins in transcriptional network at the threshold of 0.015.

**Heat shock proteins**	**Type**	**Network at heat shock of 37°C**	**Network at heat shock of 48°C**
AFU1G11180	Hsp78		AFU6G06460(+)
AFU3G14540	Hsp30/42	AFU1G12530(-), AFU2G11260(+), AFU3G01450(+), AFU3G14590(+), AFU4G08170(+), AFU4G12010(+), AFU5G04170(+), AFU6G06430(+), AFU6G06470(+), AFU6G07720(+), AFU6G08750(+), AFU6G10650(-), AFU6G10660(-), AFU7G00170(-), AFU7G00200(-), AFU7G01000(+)	AFU6G06460(+), AFU6G06470(+)
AFU5G04170	Hsp90	AFU3G14540(+), AFU6G06430(+), AFU6G06470(-), AFU6G10610(-), AFU6G10650(-), AFU7G01000(-), AFU7G00120(-), AFU7G00170(-)	AFU6G06460(+)
AFU6G06470	Hsp30	AFU1G11190(+), AFU1G12390(-), AFU1G15590(-), AFU1G16530(-), AFU2G01860(+), AFU2G10930(+), AFU3G00880(+), AFU3G08470(-), AFU3G10480(-), AFU3G14420(+), AFU3G14540(+), AFU4G08110(+), AFU4G11730(+), AFU5G03800(+), AFU5G04170(-), AFU6G06430(+), AFU6G07470(+), AFU6G08580(+), AFU6G08750(+), AFU6G10610(-), AFU6G10650(-), AFU7G00100(-), AFU7G00120(-), AFU7G00170(-), AFU7G00450(+), AFU8G01800(+)	AFU1G03150(+), AFU1G03650(-), AFU1G07420(+), AFU1G10610(-), AFU1G11130(+), AFU1G11190(+), AFU1G15380(+), AFU1G17070(+), AFU2G00180(+), AFU2G00580(+), AFU2G02000(+), AFU2G03030(+), AFU2G04010(+), AFU2G05000(+), AFU2G08930(-), AFU2G10900(+), AFU3G00130(+), AFU3G00900(+), AFU3G03140(-), AFU3G03310(+), AFU3G04120(+), AFU3G08100(-), AFU3G09360(-), AFU3G09670(+), AFU3G10840(-), AFU3G12600(+), AFU3G12620(+), AFU3G14080(+), AFU3G14500(+), AFU3G14540(+), AFU3G14950(-), AFU4G06870(-), AFU4G07330(-), AFU4G08230(+), AFU4G08340(-), AFU4G09600(-), AFU4G10000(+), AFU4G11580(+), AFU4G12670(-), AFU5G02320(-), AFU5G02840(-), AFU5G03250(+), AFU5G04100(+), AFU5G08750(-), AFU5G09020(+), AFU5G11040(+), AFU6G03050(+), AFU6G03660(+), AFU6G06460(+), AFU6G07990(+), AFU6G08750(+), AFU6G10450(+), AFU6G10650(-), AFU6G11410(+), AFU6G11430(-), AFU6G11890(+), AFU6G13500(+), AFU7G00950(+), AFU7G01920(-), AFU7G05840(+), AFU7G07100(-), AFU8G02090(+), AFU8G04840(+), AFU8G06340(-), AFU8G06350(-)

The underlined genes are metabolic genes and the signs '+' and '-' in the parenthesis represent positive and negative associations with heat shock protein, respectively.

**Figure 7:**
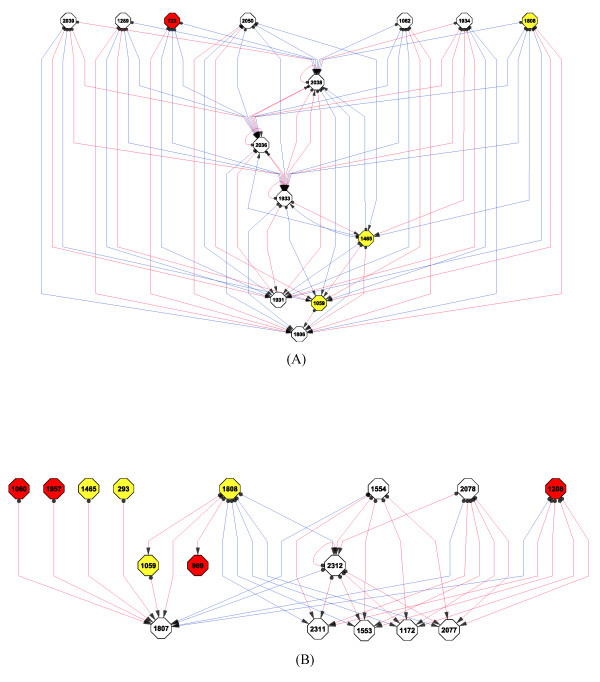
**The extracted regulation network from Figure 5 consisting of heat shock proteins, transcription factors and hub metabolic genes**. The gene corresponding to each node index is the same as Figure 5. The red and yellow nodes represent transcription factor and heat shock protein, respectively. (A) Regulation structure at heat shock of 37°C, (B) Regulation structure at heat shock of 48°C.

**Figure 8:**
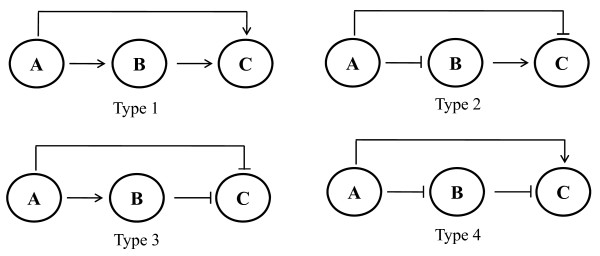
**Types of three-node coherent feed-forward loop**. Arrow and ⊣ symbol denote positive and negative regulation, respectively.

To more clarification of the regulation structure between heat shock proteins and hub metabolic genes, we extracted a small network including heat shock proteins, hub metabolic genes and transcription factors from network of Figure 5. The heat shock protein 1808 and a metabolic gene 2038 show coherent feed-forward loop (FFL) type of regulation against metabolic gene 1933 (Figure 7A). The FFL is composed of first regulator that regulates a second regulator, and both regulators regulate the same target. In this network, the 1808 negatively regulates both 1933 and 2036 where 2036 again positively regulates 1933. The indirect regulation of 1808 towards 1933 through 2036 gives finally the same effect of negative regulation by 1808 towards 1933. Thus, the expression of 1933 will be easily downregulated by both regulations of 1808 and 2036. The coherent FFL type of regulation is also shown in the network of 48°C (Figure 7B). There are four types of coherent FFL in network including 3 nodes (Figure 8). The network in Figure 7 shows that the heat shock protein 1808 employs the type 2 of coherent FFL which can act as a response accelerator for the regulation of target genes. This indicates that heat shock protein rapidly and efficiently regulates target genes by the type 2 of coherent FFL with hub metabolic genes. Considering a FFL including first regulator 1808, second regulator 2036 and target 1933 (Figure 7A), the second regulator 2036 shows positive auto-regulation. A positive auto-regulation gives slow response time in expression change, but the state of once changed expression can be maintained, even after its regulator has vanished [15]. This type of positive auto-regulation might help the cells of *A. fumigatus *to keep the heat shock mode for a time after removal of heat shock.

The regulation of transcription factor 723 (AFU3G02000, cutinase transcription factor 1*β*) at network of 37°C towards other genes may be result of indirect regulation through other intermediate genes hidden. Unlikely to the network of 37°C, many transcription factors appeared at the network of 48°C including 909 (AFU3G09670, C6 transcription factor (*oefC*)), 1060 (AFU3G14550, RNA polymerase II transcription factor B subunit 5), 1208 (AFU4G09710, C6 sexual development transcription factor (*NosA*)) and 1957 (AFU6G12160, C6 transcription factor) (Figure 5B). 909 and 1208 were downregulated while 1060 and 1957 were strongly upregulated during time course at the heat shock of 48°C (see additional file 2). The relationship of these transcription factors with other metabolic genes might be the reflection of indirect interaction rather than direct regulation through other intermediate genes hidden. The expression patterns of the C6 sexual development transcription factor 1208 and heat shock protein 1808 show clear contrast. This suggests that the positive regulation by 1208 towards a target gene brings the same effect as the negative regulation by 1808 towards the same target gene. In fact, half of metabolic genes negatively regulated by 1808 are positively regulated by 1208 (Figure 7B).

The absence of heat shock transcription factor in the networks is due to its lower interaction value with other genes than threshold. Both heat shock conditions, the heat shock transcription factor (AFU5G01900) showed upregulation like most of heat shock proteins during time course (see additional file 2). The metabolic genes in two extracted networks of 37°C and 48°C are different, but the FFL type 2 of regulation structure including heat shock proteins and metabolic genes is well-conserved in both networks. This suggests that the FFL type 2 of regulation structure is important network motif in the thermal adaptation process. The frequency of this network motif decreased with increasing temperature. That is, the FFL type 2 of regulation structure was more frequent at network of 37°C than at network of 48°C. This indicates that the regulation efficiency of heat shock proteins would be reduced at heat shock of 48°C, which might be related to dramatic increase in the expression level of heat shock proteins and the number of heat shock response genes at heat shock of 48°C.

## Discussion

It is widely accepted that biological systems are composed of functional modules with specific combinations of genes and proteins responsible for different biological functions [16,17]. The module can be considered as the temporal activity of a group of genes/proteins that controls a specific function in different environmental conditions. In this study, we identified the transcriptional module of heat response genes of *A. fumigatus *using SSM with the state dimension of *k *= 2. The genes with similar expression profile are successfully assigned to the same module. The estimated gene regulation structure based on identified module showed clearly the negative relationship between heat shock proteins and metabolic genes. Our analysis suggests that the metabolic genes as well as heat shock proteins might be used as key regulator in the thermal adaptation process of *A. fumigatus*. Especially, the metabolic genes with opposite expression pattern of heat shock proteins have a propensity to be involved in coherent FFL type 2 of regulation with heat shock proteins in the transcriptional network (Figure 5, Figure 6 and Figure 7).

The coherent FFL type 2 of regulation could make it possible to rapidly and efficiently adapt the cells of *A. fumigatus *to the change of temperature. For example, small amount of heat shock proteins can efficiently regulate the target genes. However, this type of regulation at heat shock of 48°C seems to be weak due to the reduction of metabolic genes showing coherent regulation with heat shock proteins. This might be related to dramatic increase in the amount of heat shock proteins and the number of heat shock response genes at heat shock of 48°C. Takemoto *et al*. [13] surveyed the dependence of the structure of metabolic networks in prokaryotes on their growth temperature. They found that the edge density becomes small, the clustering-coefficient-based modularity of the networks become small, and the frequency of recurring sub-graphs decays with increasing growth temperature. Although these findings are concerned with metabolic network, they show a good agreement with our results in terms of network structure. Besides edge density, the decrease of coherent FFL type 2 of regulation including heat shock protein at 48°C could be related to the fact that the frequency of recurring sub-graphs or network motifs decays with increasing temperature. Thus, the thermal adaptation process of *A. fumigatus *could be considered as change of network structure such as transition from metabolic network of mesophile into that of thermophile. This suggests that the network of 48°C might be optimal in the temperature for the survival of *A. fumigatus *even if it looks like less efficiently than the network of 37°C. Especially, in heat shock of 48°C many pathogenic genes such as AFU4G09580 (major allergen Asp F2) and AFU6G02280 (Asp F3) are appeared as heat response genes and these genes are upregulated at all time points except for 120 min (see additional file 2). This indicates that the cells of *A. fumigatus *at heat shock of 48°C might be more pathogenic than those at heat shock of 37°C.

Our results suggest for the first time that the thermal tolerance of *A. fumigatus *might be due to the efficient regulation of metabolic genes by heat shock proteins taking coherent FFL type 2 of regulation structure. We analysed the network at a fixed threshold of 0.015, but this type of regulation was well conserved at various thresholds. Thus, the network analysed in this study would be sufficient for the capturing transcriptional regulation characteristic of *A. fumigatus *in the thermal adaptation process.

## Conclusion

The understanding of thermal adaptation mechanism in the opportunistic pathogen *A. fumigatus *is important in fungal infectiology. We estimated the transcriptional regulation structure of *A. fumigatus *under heat shock conditions using time series microarray data with very short length of time point, *i.e*., 6. We propose a thermal adaptation mechanism of *A. fumigatus *through heat shock proteins, which might efficiently regulate their target genes using the coherent FFL type 2 of regulation structure. This type of regulation structure would also be efficient cellular strategy for other stressful conditions requiring rapid change of metabolic mode.

## Methods

### Pre-processing of time series microarray data

**Figure 9:**
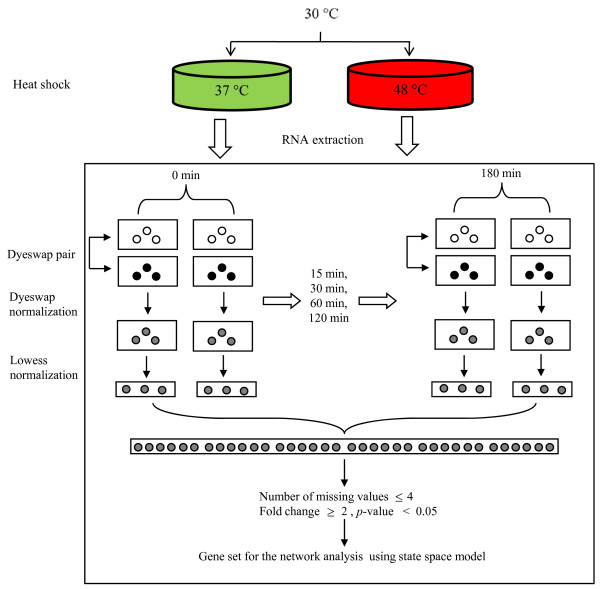
**The schematic diagram for pre-processing of microarray data**.

The time series microarray data of *A. fumigatus *for heat shock response were downloaded from ArrayExpress (, accession id: E-MEXP-332). The gene expression was examined at six time intervals including 0, 15, 30, 60, 120 180 min after shift of growth temperatures from 30°C to 37°C and 48°C, respectively. Two pairs of dye-swap arrays, *i.e*., four arrays, are used for each time point. To remove biases that arise from microarray, we conducted two-stage normalization, *i.e*., dye-swap normalization and *lowess *normalization. After dye-swap normalization, each time point has two arrays (Figure 9). For the successful application of SSM to this short time-series microarray data, we utilized all the replicates on the array for the parameter estimation of SSM. Thus, total 36 observations were obtained for each gene (triplicate on each array by two array by six time point) and the genes with more than 4 missing values were excluded (Figure 9). We selected the significantly differentially expressed genes showing over two fold change in expression level and below 0.05 of *p*-value in *t*-test for at least a time point, called heat shock response genes here. Finally, the number of heat shock response gene became 726 and 2200 for heat shock of 37°C and 48°C, respectively. The regulation structure of these heat shock response genes was estimated by our SSM. The assignment of *A. fumigatus *genes to KEGG metabolic pathways was done using the Bioconductor package KEGGSOAP . To visualize the estimated network, we used Cytoscape 2.6.0 .

### State space model

**Figure 10:**
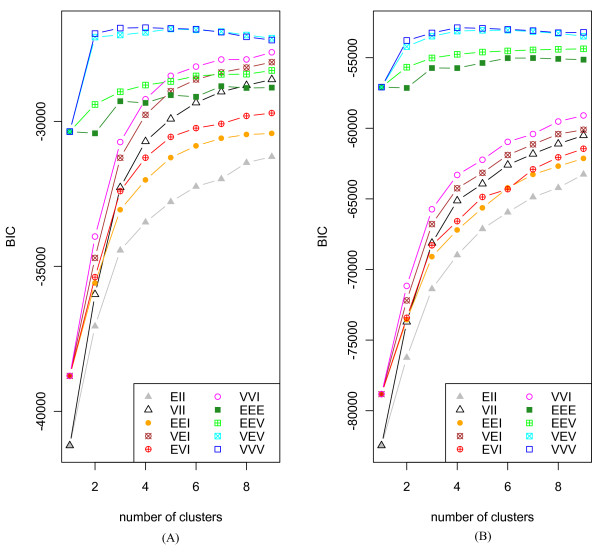
**The BIC curves for the model-based clustering of microarray data including heat response genes**. (A) BIC curve for model-based clustering of gene expression data at 37°C, (B) BIC curve for model-based clustering of gene expression data at 48°C. The numerical values of BIC in each model are shown at additional file 4. The model is divided according to the covariance matrix equation form. EII: spherical, equal volume; VII: spherical, unequal volume; EEI: diagonal, equal volume and shape; VEI: diagonal, varying volume, equal shape; EVI: diagonal, equal volume, varying shape; VVI: diagonal, varying volume and shape; EEE: ellipsoidal, equal volume, shape, and orientation; EEV: ellipsoidal, equal volume and equal shape; VEV: ellipsoidal, equal shape; VVV: ellipsoidal, varying volume, shape, and orientation.

The SSM employed in this study has been reported in our previous paper [12]. Here, we give brief description of this model. Let *y*_*n *_∈ *R *^*p*^, *n *∈ *N*_*obs *_⊆ *N *be a series of vectors containing observed expression levels of *p *genes at the *n*th time points. A sequence of the observation vectors, , is modeled by supposing that *y*_*n *_is generated from *k*-dimensional hidden state variable denoted by *x*_*n*_. To determine the dimension of state variable, we calculated the Bayesian information criterion (BIC) value across *k *= 1 to 9. However, The BIC curves were monotonically decreasing with increasing state dimension, which makes it difficult to determine the optimal dimension. Such a tendency becomes prominent when the number of sample is much smaller than the dimension of data. Thus, we directly surveyed regulation relationship between modules or sub-modules for each dimension using heat map such as the upper part of Figure 3. In this study, the dimension of state variable was chosen by *k *= 2 because regulation relationship between sub-modules was most clearly shown at this dimension. This implies implicitly that the time series microarray data of heat shock response genes might be divided into four groups because each component of state variable is divided into two sub-modules which usually have opposite pattern. The clustering analysis using the R package mclust  showed that the optimal cluster number for both datasets of 37°C and 48°C is four, where the highest BIC value is observed (Figure 10 and additional file 4). This supports our choice of *k *= 2.

We consider a linear-Gaussian SSM:

(1)

(2)

where *F *∈ *R*^*k *× *k *^is the state transition matrix, *H *∈ *R*^*p *× *k *^is the observation matrix, *v*_*n *_~ *N*_*k *_(0_*k*_, *Q*) and *w*_*n *_~ *N*_*p*_(0_*p*_, *R*) are the system noise and the observation noise, respectively. The initial state vector *x*_0 _is assumed to be a Gaussian random vector with mean vector *μ*_0 _and covariance matrix Σ_0_. For the inference of gene regulatory system underlying the observation data, the parameters *θ *= {*H*, *F*, *Q*, *R*, *μ*_0_} ∈ Θ were estimated by EM algorithm with a fixed *k*. For the identifiability of the estimated model, we used following constraints on the parameters:



Imposement of an arbitrary sign on the elements of the first row of *H*.

Thus, the parameters are reduced to *θ *= {*H*, *F*, *R*, *μ*_0_}. The estimation of these parameters is limited when the length of time series is very short, *e.g*., less than 10. This limitation can be overcome if replicates of time-course gene expression profiles are available. The dataset in each heat shock condition consists of six time points with six replicates (see pre-processing of time series microarray data in Methods section). Thus, we incorporate all replicates into parameter estimation. For this, we assume that each of the replicated time-courses is independently and identically distributed according to

(3)

(4)

where  and  represent the gene expression vector which is measured by the *l*-th replicate and the corresponding state vector at time *n*, respectively. The total number of replicates is denoted by *m *(*l *= 1,...,*m*). Given this generative model, the parameter estimation amounts to maximizing the likelihood function l(*θ*) over *θ*:

(5)

where  and . The maximum likelihood is calculated by modified EM algorithm [12]. After parameter estimation, gene expression vectors  can be mapped onto the state space *R*^*k *^with the projection matrix *D *∈ *R*^*k *× *p *^by transforming the observation model (equation 4) under above constraints as follows:

(6)

where the projection matrix is parameterized as

(7)

If the state dimension *k *is less than *p*, the dimensionality of the noise-removed gene expression vectors  is reduced by the semi-orthogonal projection matrix *D*.

During the parameter estimation process, the reduced-rank data (equation 6) are possibly constructed such that they are likely follow first-order Markov process of equation 3. This process automatically discovers *k *modules of genes that are relevant to the temporal structure of gene expressions. We can choose groups of genes, *i.e*., modules using the projection matrix *D *of which element *d*_*ij *_represents the contribution of the *j*th gene to the *i*th coordinate of the state variable, . The rank of |*d*_*ij*_| for a fixed *i *can be used for selection of genes belonging to the *i*th module from gene list. That is, genes with high contribution to the *i*th coordinate of state variable would be top-ranked and selected as the *i*th module. A module can also be divided two sub-module by the signs of *d*_*ij *_and the sub-module is determined using a similar approach to the module determination. The dynamic interactions between modules can be obtained through the system model (equation 3). Because it describes effects from  to , the relationship can be seen causal relationship. Thus, we can expect to obtain module-regulatory networks by using the estimated parameter, *i.e*., *F*. For the estimation of gene regulatory network using parameters obtained above, we converted the SSM (equations 3 and 4) to a parsimonious representation of the first order vector autoregressive form as below



where the autoregressive coefficient matrix is given by Ψ ≡ *D*^*T*^Λ*FD*. The gene regulatory network was estimated based on Ψ which represents magnitude of interactions between genes.

## Authors' contributions

JHD conceived the study, carried out data analysis, and wrote the manuscript. RY provided his valuable experience in SSM and reviewed the manuscript. SM supervised the application of SSM to identification of transcriptional network. All authors read and approved the final the manuscript.

## Supplementary Material

Additional file 1 **The thermal response of each metabolic pathway in *A. fumigatus***. This document contains the number of heat shock response genes appeared in each metabolic pathway at 15, 30, 60,120 and 180 min after heat shock. Click here for file

Additional file 2 **Average gene expression profiles of heat response genes**. This Exel file contains average gene expression profiles of heat response genes in two heat shock conditions of 37°C and 48°C. Click here for file

Additional file 3 **The node index and its corresponding gene**. This Exel file contains the gene information corresponding to each node in network estimated by state space model. Click here for file

Additional file 4 **BIC values for model-based clustering of gene expression profiles**. This Exel file contains the BIC values obtained by model-based clustering with the R package mclust for two heat shock datasets of 37°C and 48°C. Click here for file
