# Supplementary material for: Exploring temporal transcription regulation structure of Aspergillus fumigatus in heat shock by state space model
Source: BMC Genomics. 2009 Jul 8;10:306. doi: 10.1186/1471-2164-10-306 (PMC2714559; doi:10.1186/1471-2164-10-306)
Supplement: Additional file 1 — The thermal response of each metabolic pathway in A. fumigatus. This document contains the number of heat shock response genes appeared in each metabolic pathway at 15, 30, 60,120 and 180 min after heat shock. [file 1471-2164-10-306-S1.doc]

**The number of heat shock response genes appeared in each metabolic pathway.**

|  | Heat shock of 37 °C | | | | | | | | | | Heat shock of 48 °C | | | | | | | | | |
| --- | --- | --- | --- | --- | --- | --- | --- | --- | --- | --- | --- | --- | --- | --- | --- | --- | --- | --- | --- | --- |
| Metabolic pathway | 15 min | | 30 min | | 60 min | | 120 min | | 180 min | | 15 min | | 30 min | | 60 min | | 120 min | | 180 min | |
| (No. of metabolic genes) | **+** | **-** | **+** | **-** | **+** | **-** | **+** | **-** | **+** | **-** | **+** | **-** | **+** | **-** | **+** | **-** | **+** | **-** | **+** | **-** |
| Glycolysis /Gluconeogenesis (52) | 0 | 1 | 0 | 2 | 2 | 2 | 6 | 1 | 2 | 0 | 4 | 4 | 7 | 11 | 6 | 10 | 6 | 11 | 12 | 9 |
| Citrate cycle (29) | 0 | 2 | 0 | 2 | 8 | 2 | 11 | 2 | 4 | 1 | 0 | 4 | 0 | 13 | 0 | 12 | 11 | 3 | 8 | 4 |
| Pentose phosphate pathway (27) | 0 | 2 | 0 | 0 | 1 | 2 | 1 | 1 | 0 | 0 | 4 | 1 | 3 | 5 | 1 | 3 | 4 | 3 | 5 | 4 |
| Fructose and mannose metabolism (94) | 0 | 1 | 0 | 1 | 3 | 0 | 3 | 1 | 1 | 2 | 4 | 3 | 3 | 9 | 5 | 8 | 4 | 7 | 6 | 10 |
| Galactose metabolism (35) | 0 | 0 | 0 | 1 | 0 | 0 | 1 | 1 | 0 | 0 | 1 | 2 | 1 | 4 | 1 | 3 | 1 | 3 | 1 | 2 |
| Stach and sucrose metabolism (78) | 4 | 0 | 1 | 1 | 0 | 2 | 1 | 2 | 0 | 1 | 7 | 2 | 7 | 5 | 5 | 3 | 3 | 2 | 3 | 4 |
| Aminosugars metabolism (110) | 1 | 3 | 1 | 0 | 1 | 1 | 1 | 3 | 2 | 4 | 2 | 4 | 2 | 8 | 2 | 10 | 2 | 8 | 4 | 12 |
| Pyruvate metabolism (43) | 0 | 2 | 1 | 3 | 4 | 3 | 7 | 2 | 5 | 1 | 0 | 4 | 2 | 8 | 3 | 8 | 12 | 6 | 10 | 7 |
| Propanoate metabolism (50) | 1 | 1 | 0 | 1 | 1 | 1 | 6 | 1 | 2 | 1 | 1 | 1 | 2 | 3 | 4 | 4 | 6 | 2 | 11 | 1 |
| Oxidative phosphorylation (75) | 1 | 0 | 0 | 0 | 2 | 0 | 5 | 0 | 1 | 0 | 0 | 7 | 0 | 15 | 0 | 18 | 4 | 11 | 4 | 10 |
| Fatty acid metabolism (37) | 0 | 0 | 0 | 0 | 2 | 0 | 5 | 0 | 3 | 0 | 2 | 0 | 4 | 0 | 5 | 3 | 4 | 4 | 8 | 3 |
| Glycerophospholipid metabolism (53) | 1 | 0 | 0 | 1 | 1 | 1 | 2 | 0 | 1 | 1 | 3 | 4 | 2 | 7 | 3 | 4 | 2 | 3 | 5 | 5 |
| Purine metabolism (86) | 0 | 12 | 0 | 0 | 0 | 1 | 0 | 3 | 0 | 0 | 4 | 13 | 4 | 13 | 2 | 12 | 6 | 5 | 5 | 6 |
| Pyrimidine metabolism (61) | 0 | 8 | 1 | 1 | 0 | 0 | 0 | 0 | 0 | 0 | 3 | 7 | 4 | 5 | 2 | 5 | 1 | 3 | 1 | 2 |
| Glutamate metabolism (37) | 0 | 3 | 3 | 2 | 4 | 0 | 5 | 2 | 1 | 1 | 3 | 3 | 4 | 9 | 5 | 8 | 4 | 4 | 6 | 6 |
| Alanine and asparate metabolism (39) | 1 | 3 | 0 | 2 | 2 | 3 | 4 | 2 | 0 | 1 | 2 | 3 | 1 | 13 | 1 | 12 | 1 | 5 | 4 | 12 |
| Glycine,serine and threonine metabolism (116) | 0 | 2 | 0 | 0 | 3 | 0 | 2 | 1 | 1 | 0 | 4 | 3 | 5 | 8 | 7 | 6 | 2 | 1 | 5 | 5 |
| Valine, leucine and isoleucine synthesis (20) | 0 | 4 | 2 | 1 | 2 | 1 | 0 | 1 | 0 | 0 | 0 | 4 | 0 | 6 | 0 | 3 | 3 | 1 | 2 | 3 |
| Arginine and proline metabolism (21) | 1 | 1 | 2 | 0 | 1 | 0 | 6 | 0 | 1 | 1 | 1 | 1 | 1 | 8 | 1 | 6 | 1 | 1 | 4 | 4 |
| Histidine metabolism (42) | 0 | 1 | 0 | 0 | 1 | 0 | 2 | 1 | 1 | 0 | 1 | 2 | 2 | 4 | 2 | 3 | 3 | 1 | 4 | 0 |
| Phenylalanine, tyrosine and tryptophan biosynthesis (34) | 0 | 4 | 0 | 0 | 1 | 0 | 2 | 0 | 0 | 0 | 0 | 5 | 0 | 12 | 0 | 6 | 0 | 1 | 1 | 4 |
| Glutathione metabolism (27) | 1 | 1 | 1 | 0 | 1 | 1 | 1 | 1 | 0 | 1 | 2 | 1 | 2 | 3 | 0 | 5 | 1 | 4 | 1 | 5 |
| N-Glycan biosynthesis (38) | 0 | 1 | 0 | 0 | 1 | 0 | 0 | 0 | 0 | 0 | 0 | 4 | 2 | 5 | 0 | 2 | 0 | 2 | 1 | 5 |
| Glycan structures - Biosynthesis 1 (30) | 0 | 1 | 0 | 1 | 1 | 0 | 0 | 0 | 0 | 0 | 0 | 8 | 0 | 8 | 0 | 5 | 0 | 1 | 1 | 5 |
| Glycan structures - Biosynthesis 2 (16) | 1 | 2 | 0 | 0 | 0 | 0 | 0 | 0 | 0 | 0 | 0 | 4 | 0 | 3 | 0 | 1 | 0 | 0 | 0 | 2 |
| Riboflavin metabolism (30) | 0 | 2 | 0 | 0 | 0 | 0 | 1 | 1 | 0 | 3 | 1 | 2 | 1 | 5 | 0 | 4 | 1 | 3 | 1 | 5 |
| Folate biosynthesis (49) | 0 | 4 | 0 | 0 | 0 | 2 | 1 | 0 | 0 | 1 | 2 | 4 | 2 | 6 | 2 | 2 | 2 | 0 | 3 | 2 |
| Ubiquinone biosynthesis (11) | 0 | 0 | 0 | 1 | 0 | 0 | 0 | 0 | 0 | 0 | 1 | 0 | 0 | 0 | 0 | 3 | 0 | 4 | 0 | 3 |
| Alkaloid biosynthesis II (49) | 1 | 0 | 0 | 1 | 1 | 0 | 2 | 0 | 0 | 0 | 0 | 2 | 0 | 4 | 0 | 3 | 1 | 3 | 4 | 5 |
| RNA polymerase (25) | 0 | 4 | 0 | 0 | 0 | 0 | 0 | 0 | 0 | 0 | 1 | 2 | 1 | 0 | 1 | 0 | 1 | 0 | 1 | 0 |
| Basal transcription factors (20) | 0 | 0 | 0 | 0 | 0 | 0 | 0 | 0 | 0 | 0 | 2 | 0 | 4 | 0 | 5 | 0 | 1 | 0 | 2 | 0 |
| Ribosome(76) | 0 | 11 | 0 | 4 | 0 | 0 | 0 | 23 | 0 | 0 | 1 | 28 | 1 | 48 | 0 | 47 | 0 | 2 | 1 | 33 |
| Aminoacyl-tRNA biosynthesis (39) | 1 | 3 | 0 | 0 | 0 | 1 | 0 | 1 | 0 | 0 | 1 | 8 | 0 | 13 | 0 | 4 | 0 | 0 | 2 | 1 |
| Ubiquitine mediated proteolysis (50) | 0 | 0 | 0 | 0 | 0 | 0 | 1 | 1 | 3 | 0 | 5 | 4 | 6 | 4 | 8 | 2 | 4 | 2 | 5 | 1 |
| proteasome (31) | 0 | 0 | 0 | 1 | 0 | 0 | 0 | 0 | 0 | 0 | 0 | 2 | 0 | 7 | 0 | 1 | 0 | 2 | 0 | 0 |
| Nucleotide excision repair (36) | 0 | 0 | 0 | 0 | 0 | 0 | 0 | 0 | 0 | 1 | 1 | 4 | 3 | 1 | 2 | 2 | 0 | 1 | 2 | 2 |
| Cell cycle (yeast) (61) | 0 | 0 | 0 | 0 | 0 | 0 | 0 | 1 | 1 | 0 | 2 | 6 | 3 | 8 | 4 | 4 | 0 | 1 | 3 | 2 |

The metabolic genes are based on KEGG data base (http://www.genome.jp/kegg) (The signs of ‘+’ and ‘-’ represent upregulation and downregulation, respectively). The upregulation of metabolic genes in TCA cycle begins to appear at 60 min (37 °C) and 120 min (48 °C ) after heat shock.
